# Possible role of gut microbes and host’s immune response in gut–lung homeostasis

**DOI:** 10.3389/fimmu.2022.954339

**Published:** 2022-10-04

**Authors:** Sonakshi Rastogi, Sneha Mohanty, Sapna Sharma, Prabhanshu Tripathi

**Affiliations:** ^1^ Food Drug and Chemical Toxicology Division, Council of Scientific and Industrial Research (CSIR)-Indian Institute of Toxicology Research, Lucknow, Uttar Pradesh, India; ^2^ Institute of Biosciences and Biotechnology, Shri Ramswaroop Memorial University, Barabanki, Uttar Pradesh, India

**Keywords:** gut–lung axis, gut microbiome, airway microbiome, diet, short-chain fatty acids, lung immunity, bibliographic analysis

## Abstract

The vast diversity of microbial communities reside in various locations of the human body, and they are collectively named as the ‘Human Microbiota.’ The majority of those microbes are found in the gastrointestinal and respiratory tracts. The microorganisms present in the gastrointestinal and the respiratory tracts are called the gut microbiota and the airway microbiota, respectively. These microbial communities are known to affect both the metabolic functions and the immune responses of the host. Among multiple factors determining the composition of gut microbiota, diet has played a pivotal role. The gut microbes possess enzymatic machinery for assimilating dietary fibers and releasing different metabolites, primarily short-chain fatty acids (SCFAs). The SCFAs modulate the immune responses of not only the gut but other distal mucosal sites as well, such as the lungs. Dysbiosis in normal gut flora is one of the factors involved in the development of asthma and other respiratory disorders. Of note, several human and murine studies have indicated significant cross-talk between gut microbiota and lung immunity, known as the gut–lung axis. Here, in this review, we summarize the recent state of the field concerning the effect of dietary metabolites, particularly SCFAs, on the “gut–lung axis” as well as discuss its impact on lung health. Moreover, we have highlighted the role of the “gut–lung axis” in SARS-CoV-2 mediated inflammation. Also, to analyze the global research progress on the gut–lung axis and to identify the knowledge gap in this field, we have also utilized the bibliographic tools Dimension database and VOS viewer analysis software. Through network mapping and visualization analysis, we can predict the present research trend and the possibility to explore new directions.

## Introduction

The vast diversity of microbial communities reside symbiotically on and within various sites of the human body, like the skin, gastrointestinal tract, oral cavity, respiratory tract, and genital organs; these are collectively known as the “Human Microbiota” (1). The term “microbiota” refers to microbes in a given habitat, whereas the term “microbiome” is used when their genes are also under consideration. Most of the literature uses them interchangeably, and we are also using both terms interchangeably in the review. The microorganisms present in the gastrointestinal and the respiratory tract are known as the gut microbiota and the airway microbiota, respectively (2, 3). These microorganisms belong to archaea, protozoa, viruses, eukaryotes, and predominantly bacteria, which are known to regulate the immune responses and metabolic functions in our body (1). The composition of the gut microbiota is largely determined by the diet (4). The gut microbes possess enzymatic machinery for assimilating dietary fibers which are indigestible by humans, releasing different metabolites as a by-product (5). Emerging evidence supports the role of these microbial metabolites in tuning the immune system of the host (innate as well as adaptive) to maintain the homeostasis between tolerance and response (6). Among metabolites, short-chain fatty acids (SCFAs) are the most extensively studied and are reported to modulate the immune response of not only the gut but other distal mucosal sites as well, such as the lungs (7) Any disturbance in the dynamics of the host–microbe relationship mediated by SCFA is associated with a multitude of health conditions affecting both the gut and the lung, such as asthma, allergy, and cystic fibrosis (8). Micro-aspiration of gut microbes or mobilization of activated immune components *via* the bloodstream or lymph can also affect lung immune responses (9). Dysbiosis in normal gut microbiota is one of the important factors in the consideration of the development of asthma and other respiratory disorders. Of note, several human and murine studies indicate a significant connection between gut microbiota and lung immunity, known as the gut–lung axis (10). Here, in this review, we have summarized the recent state of the field concerning the effect of dietary metabolites, particularly SCFAs, on the “gut–lung axis” as well as discussed its impact on lung health. Besides, we have highlighted the role of the “gut–lung axis” in virus-mediated inflammation such as SARS-CoV-2 infection.

**Figure 1:**
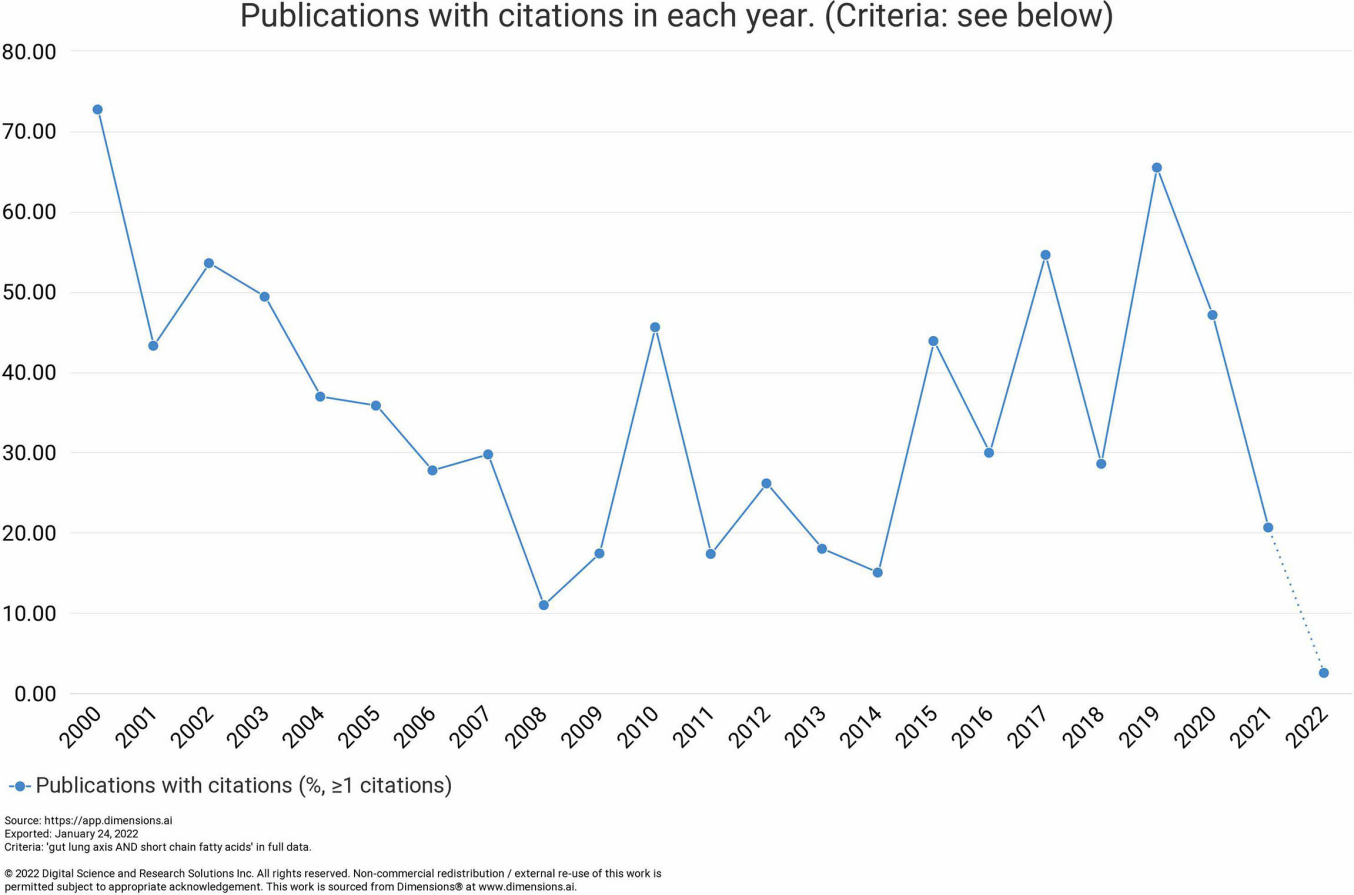
Year-wise trend in the publication for the last 20 years (2003–2022).

**Figure 2:**
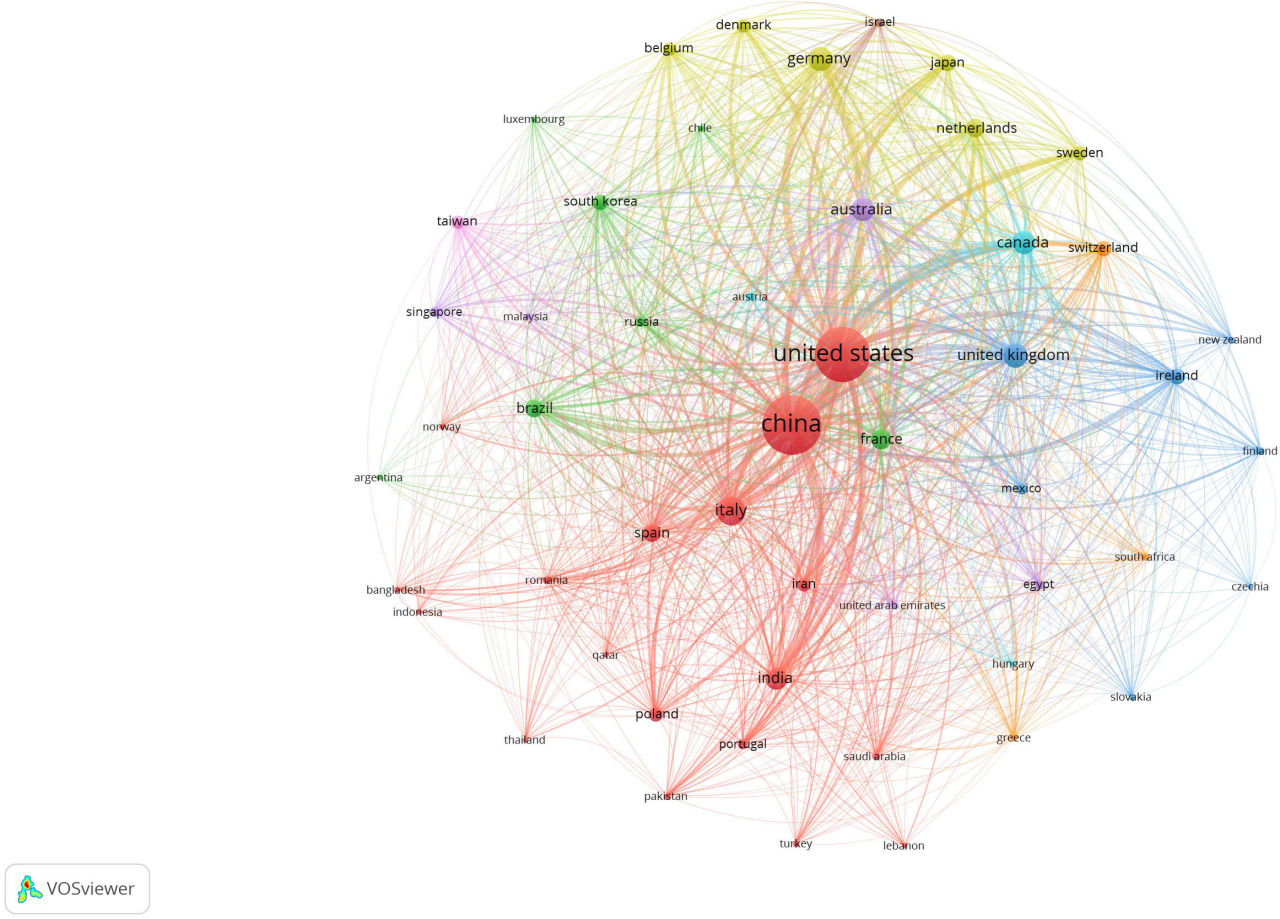
Network visualization map showing bibliographic coupling of the countries conducting research in the field of gut–lung axis and SCFAs collectively. Connecting line thickness is directly proportional to the coloration strength among the countries. The percentage of association is presented by node and its size. The network map was generated using VOS viewer visualization software.

In order to analyze the research progress on gut-lung axis research and to identify the knowledge gap in this field, we exploited the bibliographic tools available online. Published articles on this subject were collected based on an advanced search system with a defined search strategy using keywords “gut–lung axis” and “SCFAs” from the dimension database. A total number of publications were obtained from the dimension database on 4 February 2021. Results demonstrated that a total of 6,656 publications, seven grants, 501 patents, and two clinical trials were conducted on this topic in the last 20 years (2003–2022) as observed in **Figure 1**. Assessing the research activities in the last 20 years, clearly proves the growing interest of researchers worldwide in the gut-lung axids with progressive increase in publications (2017—384 publications, 2018—826 publications, 2019—784 publications, 2020—999 publications, and 2021—1,757 publications) in the last 5 years. After importing the data retrieved from the Dimension database, further bibliometric analysis was done using the VOS viewer visualization tool step by step as follows: import the source file, adjust the time slicing, set up the selection criteria and unit of analysis for each analysis. Moreover, the co-citation analysis identifies the relatedness of items and is determined based on the number of times they were cited together. Furthermore, the cluster analysis helps to recognize the structure of research, such as countries or institutes of this research. Using clustering statistics, the proximity of the relationship among items (frequency of co-occurrence within the document) can be determined. These similar relationships are collected to form clusters whose views are independent. In the present study, cluster analysis was performed based on both co-authorship and citation analysis using the VOS viewer. For co-authorship analysis, every circular circle is called a node and represents a research article, and the lines that link nodes represent the co-reference. **Figure 2** represents the network visualization of bibliographic coupling among countries. The minimum number of publications for a country was set at 10. Out of 86 countries, 51 met the threshold. The network map showed countries currently working on this topic around the globe. All the countries were linked in nine clusters, with a total of 1,275 links. The frequency of citation is proportional to the thickness of the ring. The author and year of publication of the citation are labeled on that node.

**Figure 3:**
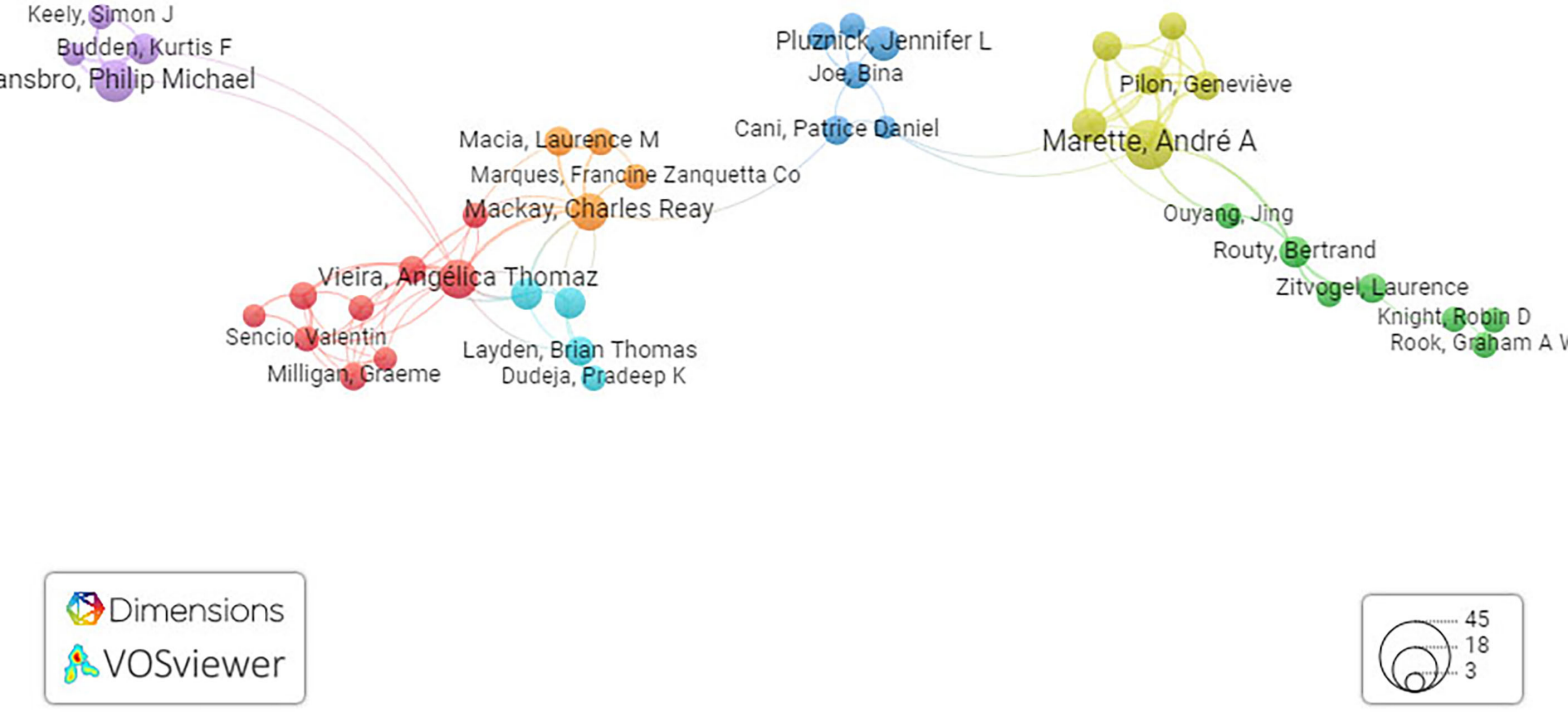
Network visualization map of co-cited references. Network visualization map of terms in published articles in literature related to the gut–lung axis and SCFAs collectively. The map shows four clusters represented in four different colors. Nodes with similar colors represent a cluster of related citations. The network map was generated using VOS viewer visualization software.

In **Figure 3**, representing the network map, it is presumed that a total of 2,828 citations with 841 citation links among 90 researchers were clustered into nine clusters worldwide. The results of this study depend on literature screened from the Dimension core collection database and an attempt being made to recollect the available literature and present it in a systematic way using the VOS viewer. Moreover, using the results obtained from network mapping and visualization analysis, we can predict the present research trend and explore new directions. This study will pave the way for research groups to identify collaborators.

## The gut microbiome

The population of commensal microbes reaches its maximal density in the gastrointestinal compartment, forming the richest ecosystem, collectively referred to as the “gut microbiota” (2). Members of gut microbiota are known to develop intricate trophic relationships among themselves and their human host; and mostly belong to *Eukarya*, *Archaea*, *Viruses*, and *Bacteria* (1). Among gut microbes, *Bacteroidetes* and *Firmicutes* are the most abundant phyla, followed by *Actinobacteria*, *Tenericutes*, *Fusobacteria*, *Proteobacteria*, and *Verrucomicrobia* (2). A growing literature with scientific evidence ascertains that the initial colonization of gut microbes start immediately after birth and is driven by various factors, primarily the feeding mode of the infant (breast-fed or formula-fed) (11). Human breast milk constitutes human milk oligosaccharides (HMOs) that selectively shape the gut microbes (12). In breast-fed infants, the taxonomic diversity of the gut include higher *Lactobacilli*, *Bifidobacteria*, *Streptococci*, and *Staphylococci*, while in formula-fed infants, more abundance of *Proteobacteria*, *Clostridia*, and *Bacteroides* was observed (12). The alterations in the compositional diversity during neonatal life have been associated with diseased states in infants that are manifested at later stages of life, such as asthma, inflammatory bowel disease, and metabolic disorders (13). Thus, it is presumed that early gut microbiota composition is responsible for disease progression in later life and that the foundation for a stable adult gut microbiota depends largely on the dietary pattern of infants (13). Early colonization activates the recruitment of several types of immune cells that in turn interact with the epithelial layer of the gut to develop a bridge capable of accumulating the microbes inside the lumen (14). The regulatory mechanisms prevent the unwanted inflammatory responses that may lead to a diseased state in the host (14). In the later stages of life, alterations in gut microbial composition depend upon multiple factors, such as diet, age, lifestyle, frequent infections, medication, hospitalizations, immune strength, and changed gut physiology (15).

Numerous reports are available that indicate that indicate appropriate symbiotic host–microbial interaction greatly contributes to host immune homeostasis and synthesis of micronutrients and vitamins such as thiamine, biotin, riboflavin, cobalamin, pantothenic acids, and nicotine, which are useful for metabolism of both the host as well as the microbe (16). In addition to these, the epithelial cells of the intestine, including goblet cells, enterocytes, and paneth cells, produce numerous antimicrobial peptides like cathelicidins, C-type lectins, and defensins that inhibit the attachment of pathogens and commensals to the gut epithelium (17). The importance of the gut microbiome in the development of mucosal immunity was established by multiple studies comparing germ free (GF) and specific pathogen free (SPF mice) and reviewed in various literature where it was found that GF mice have fewer regulatory T cells (Tregs) and Ig-A secreting plasma cells in the lamina propria (18). In a study by Dickson et al., it was shown that gut microbiota are important in shaping the immune cell repertoire of the lung by directing the development of type 3 innate lymphoid cells (ILC3) (19). Another study from mice has shown that Vancomycin driven dysbiosis in gut microbiota lead to Th2- or Th1/Th17 driven lung diseases (20). In this context, it is assumed that the microbial composition plays a significant role in the host physiology.

## The airway microbiome

Compared to the gut microbiome, studies involving airway microbiome are still limited. Recently, through next-generation sequencing techniques, it became clear that the pulmonary tract is not a sterile site and harbors distinct microbial species whose compositional diversity varies substantially between the upper and lower airways. (21). As evidenced by the prevalence of distinct genera in both the lungs and oral cavity, microbial communities in the lungs are partially seeded by microaspiration of the oral microbiome. Among them, species belonging to *Prevotella*, *Streptococcus*, and *Veillonella* are the most common (3). The predominant phyla in the oropharynx are anaerobes such as *Firmicutes*, *Spirochaetes*, *Bacteroidetes* and aerobes such as *Rothia*, *Neisseria*, *Actinomyces*, and S*treptococcus* while in the lungs is *Bacteroidetes* and *Firmicutes* among healthy individuals (9). The community composition also includes other native residents such as saprophytic protozoa, primarily *Trichomonas tenax* and *Entamoeba gingivalis* and fungi such as *Saccharomyces cerevisiae* and *Candida albicans* (22, 23).The dynamic spectrum of airway microbiota largely rely on its interaction with host diet, nutrition, immune system, genetic predisposition, recurrent infection, hormonal factors, and tobacco smoking (22). In tobacco smoking subjects, lower abundance of genus *Prevotella* and phylum *Firmicutes* (genera *Megasphaera and Veillonella*) is reported (22).

In contrast to the gut, which contains approximately 10^11^ to 10^12^ bacteria per gram of tissue, microbial biomass in healthy lungs is significantly less (10^3^ to 10^5^ bacteria per gram of tissue). Though both the respiratory and gastrointestinal tracts are covered with mucus, their micro-anatomical features and origin are distinct (24). Also, the respiratory tract is rich in oxygen (aerobic), whereas the GI tract is anaerobic (24). The temperature of the gut is constant (37°C) from esophagus to large intestine, while the temperature of the pulmonary tract has a gradient from ambient temperature to core body temperature in the lungs (24). Considering the above differential features, the gut and the airways have distinct microbial flora.

Similar to gut microbiota, the establishment of airway microbiota also starts immediately after birth and reaches to a mature compositional diversity in the first three post-natal months. (25). In healthy human lungs, the abundance of rich and diverse microbial communities is determined by the balance of three aspects: (1) removal of commensals from the airways; (2) immigration of bacteria into the airways; and (3) the relative growth rates of bacteria found in the airways that largely depend upon conditions prevailing in different regions of the respiratory tract (19). Elimination of microbes is driven by cough, host immune response, and mucociliary clearance. Ecological factors in the airways like oxygen tension, pH, nutrient availability, temperature, local microbial competition, activation of host inflammatory cells, and pulmonary epithelial cell interactions impact the airway microbiome (19, 26).

The mature airway microbiome has significant implications in development and regulation of adaptive and innate immune responses; therefore, the investigation of airway dysbiosis in the prognosis of multiple pulmonary diseases, primarily chronic obstructive pulmonary disease (COPD), asthma, interstitial respiratory disorders, and idiopathic pulmonary fibrosis is imperative. Nowadays, many research groups have focused on the comparison between the airway microbiota in healthy subjects and those with patients complaining of chronic lung diseases, primarily asthma, lung cancer, COPD, fibrosis idiopathic pulmonary disease, and cystic fibrosis (27, 28). A relative study on microorganisms present in the oral cavity and the lower airways of asthmatic and non-asthmatic patients has contributed to the characterization of microbial signatures associated with diseased states. As evidenced, the Proteobacteria phylum represented by pathogenic genera, such as *Haemophilus*, *Neisseria*, and *Moraxella*, dominated in patients with asthma as compared to non-asthmatic patients (29). Similarly, genera of *Klebsiella* that also lie within the phylum Proteobacteria were higher in subjects suffering from chronic asthma, while the phylum Actinobacteria was associated with augmentation of disease symptoms (30). These studies recognize the causal connection, which if harnessed, can be exploited as an intervention in treating asthma. Patients with severe neutrophilic asthma receive high inhaled corticosteroid (ICS) doses, leading to airway dysbiosis (31). Importantly, combined treatment of ICSs and oral glucocorticoids resulted in the enrichment of *Proteobacteria* and *Pseudomonas* species and a depletion in abundance of *Prevotella*, *Bacteroidetes*, and *Fusobacteria* in their airways (32). For instance, the phylum Actinobacteria was correlated with steroid responsiveness (FKBP5 expression) at the molecular level (30). These scientific studies propound the intricate cross-talk between the drug and the inflammatory milieu of the host in determining the airway microbial diversity of asthmatic patients on corticosteroid treatment. Studies also proved that the loss of airway microbiota diversity is correlated with indices of alveolar inflammation, affecting immunogenic, anatomic, and physiologic features of chronic respiratory diseases in the host (33). Furthermore, advanced approaches are needed to confirm the role of the microbiota of the host as biomarkers for prognosis of diseases and to decipher novel therapeutic interventions that are capable of replenishing airway dysbiosis effectively.

## Crosstalk between gut microbes and lungs

The maintenance of the immune system largely depends on gut microbial diversity, which provides metabolites that are responsible for immune system priming and maturation. Also, environmental factors, particularly antibiotic treatment, diet, and emotional stress, can change the gut microbes with reduced diversity of propitious microbial species and overgrowth of virulent strains (34). To elaborate, loss of gut–lung crosstalk is associated with increased susceptibility to airway infections and disorders, such as allergies (35). The importance of the gut–lung axis is demonstrated in patients suffering from GI tract disorders and was also found to be susceptible to disorders pertaining to the pulmonary tract (35). Studies showed a higher risk of asthma in subjects with dysbiosis. In the early life of humans, the lower abundance of bacteria belonging to *Akkermansia*, *Bifidobacteria*, and *Faecalibacteria* in the gastrointestinal tract is associated with greater chances of asthma and atopy (36). Murine and human studies suggest the importance of an early developmental stage in infants where commensals in the gut are responsible for immune response development (36). In addition to allergic airway diseases, studies have also reported the defensive action of gut microbiota against several bacterial and viral respiratory infections by regulating adaptive and innate immune responses (35). Though most of the studies focus on the single way interaction from the gut to the lung, there are chances of crosstalk in the reverse way too. Certain pulmonary disorders, such as asthma, COPD, and cystic fibrosis (CF), are linked with perturbations in both the airway and gastrointestinal microbiota (10). A pulmonary infection in mice tends to indirectly induce intestinal dysbiosis. The resulting change in gut microbiota composition induces inflammation and enteropathy through the enrichment of genus *Enterobacteriaceae* and the loss in *Lactococci* and *Lactobacilli* population (10). Thus, for maintaining homeostasis and educating the host immune response, efficient interaction between the gut and the lungs is pivotal. The detailed pathways highlighting the gut impact on lung health and vice versa are now being studied. There are multiple contributing factors that tend to exert their functional role in establishing the gut–lung axis, which includes both microbial components and their metabolites (7). Among these components, SCFAs are the most important immunomodulatory metabolites with demonstrated protective efficacy in humans suffering from airway inflammation (7). Among children at one year of age, high levels of SCFAs, particularly propionate and butyrate in feces, have been correlated with less atopic sensitization and reduced chances of developing asthma in later years of life (8). In murine models, supplementation with SCFAs is associated with a lower airway inflammatory response in murine models (7). Thus, microbial metabolites play a significant role in the amelioration of pulmonary diseases, particularly by influencing the immune responses of the host.

## SCFAs regulate immune responses

Results from several studies on laboratory animals and human cohorts have documented multiple endogenous and exogenous factors like diet, genetics, and age to impact the compositional diversity of the gut microbiota. Among the significant contributing factors, diet is one of the most appealing options for adjunct therapy in gut dysbiosis (37). Diet affects gut microbial dynamics as well as overall health, suggesting a “Diet–Microbiota–Immunity” link (4). A multitude of experimental results indicate the association of high fiber diets with reducing the levels of inflammatory markers (IL-6 and CRP) in serum (38). Regular intake of dietary fibers increases the production of bacterial metabolites, particularly; short-chain fatty acids (SCFAs) (5). These metabolites modulate the immune responses of the host and provide protection against allergic inflammation in the lungs (5). These findings emphasize the importance of diet, microbial metabolites, and gut microbiota in determining lung immune responses. Dietary fibers are unbranched and branched polysaccharides containing monosaccharide chains that are indigestible in the lumen of the small intestine of the host owing to the paucity of appropriate enzymes (39). Diverse classes of dietary fibers are present, and they differ in their ability to undergo fermentation in the gut. Soluble dietary fibers such as inulin, oligofructose, cornstarch, and psyllium have more fermentability and generate a larger quantity of SCFAs in the colonic lumen, while insoluble dietary fibers such as hemicellulose and cellulose have reduced fermentation capacity, thus producing fewer SCFAs (5). Soluble dietary fibers, with greater fermentability potential, serve as an energy source for certain groups of gut bacteria, thereby supporting their growth in the GI tract (39). One of the comparative studies conducted between European and African children, reported higher prevalence of genus *Prevotella* in the gut of African children with fiber rich diet while European children reported enrichment of the genus *Bacteroides* in their gut as they were on protein and fat rich diet (40). The fiber rich diet of African children led to enrichment of microbial species, such as *Xylanibacter* and *Prevotella*, that express genes for carrying out fermentation of fibrous components, resulting in the secretion of SCFA (e.g., propionate, acetate, and butyrate) (40). A diet enriched with fiber not only change the gut microbial diversity but also influence the airway microbiota.

In order to balance a microbe–host mutualistic relationship in the colonic lumen, the direct interaction between commensal microbes and the epithelial layer is reduced by the secretion of mucus, antimicrobial peptides, and immunoglobulin A (sIgA). The SCFAs play a very important role in maintaining and establishing mucosal immunity in the host by modulating different aspects of these defense lines. To exemplify, SCFAs were reported to promote goblet cells differentiation and mucus production, production of intestinal IgA by augmenting plasma B-cell metabolism and fortifying tight junction permeability, thereby enhancing intestinal epithelial barrier function in the gut of the host (41). In addition to this, SCFAs also sustain intestinal homeostasis by stimulating anti-inflammatory mechanisms. One of the important mechanisms of immunosuppression involves T regulatory (Treg) cells. Mice lacking Treg cell activity were more prone to the development of intestinal inflammation. The Treg cells produce high amounts of the anti-inflammatory cytokine, IL-10, that is needed for maintenance and induction of Treg cells at the molecular level (42). Oral administration of SCFAs stimulates the proliferation of Treg cells in the colonic lumen. Butyrate and propionate fortified diets in mice were found to increase the Treg cell population in the colon (43). The elevated Treg cell population was confined to the neuropillin 1-negative (Nrp1) subset, clearly highlighting the role of butyrate in the differentiation (induction) of naïve T cells into Treg. Also, butyrate elicits production of aldehyde dehydrogenase 1a (Aldh1a) and IL-10 in dendritic cells (DC) and macrophages, thereby inducing differentiation of naïve T cells into Treg cells through the GPR109A signaling pathway (42). Butyrate also suppresses colonic inflammation by inducing transcription of IL-18 (44). Mice lacking GPR109A reported reduced levels of IL-18 in their colon and are marked with higher number of *Prevotellaceae* family of bacteria as compared to wild type (WT) mice. Both GPR109Aand GPR43 activate pathways to maintain intestinal inflammation and/or gut microbiota.

In the gut, the microbial SCFAs formation leads to a reduced pH of the lumen, which results in inhibition of the growth of enteropathogens. *Bifidobacteria* produce SCFAs, particularly acetate and butyrate (45, 46). Among SCFAs, butyrate has been prominently known for its anti-inflammatory potential (6). In the gut lumen, the concentration ratio is approximately 15:25:60 butyrate (C4): propionate (C3): acetate (C2), respectively (6). The dietary fibers are fermented into butyrate in a multistep process which is mediated by anaerobic microbes in the colonic lumen, particularly belonging to *Clostridium* clusters IV and XIVa (*Roseburia intestinalis* DSM14610 and *Anaerostipescaccae* DSM14662) and *Bifidobacterium* (47). After their production as metabolites in the colon, SCFAs are transported into the colonic tissues, which is mediated by several mechanisms, such as either through diffusion, or *via* transport proteins (HCO3^−^/SCFA exchange or sodium-coupled monocarboxylate transporter-1).The presence of different types of SCFAs in the peripheral and local milieu is known to exert numerous pleiotropic functions, which include maintaining and reinforcing intestinal–epithelial integrity as well as alleviating inflammatory responses in the GI and respiratory tract of mammals (48). The understanding of how SCFAs derived from commensals associate the gut with the lung is still unexplored. However, SCFAs are known to interact either directly or indirectly with different cells, such as epithelial and lymphocytes.

**Figure 4:**
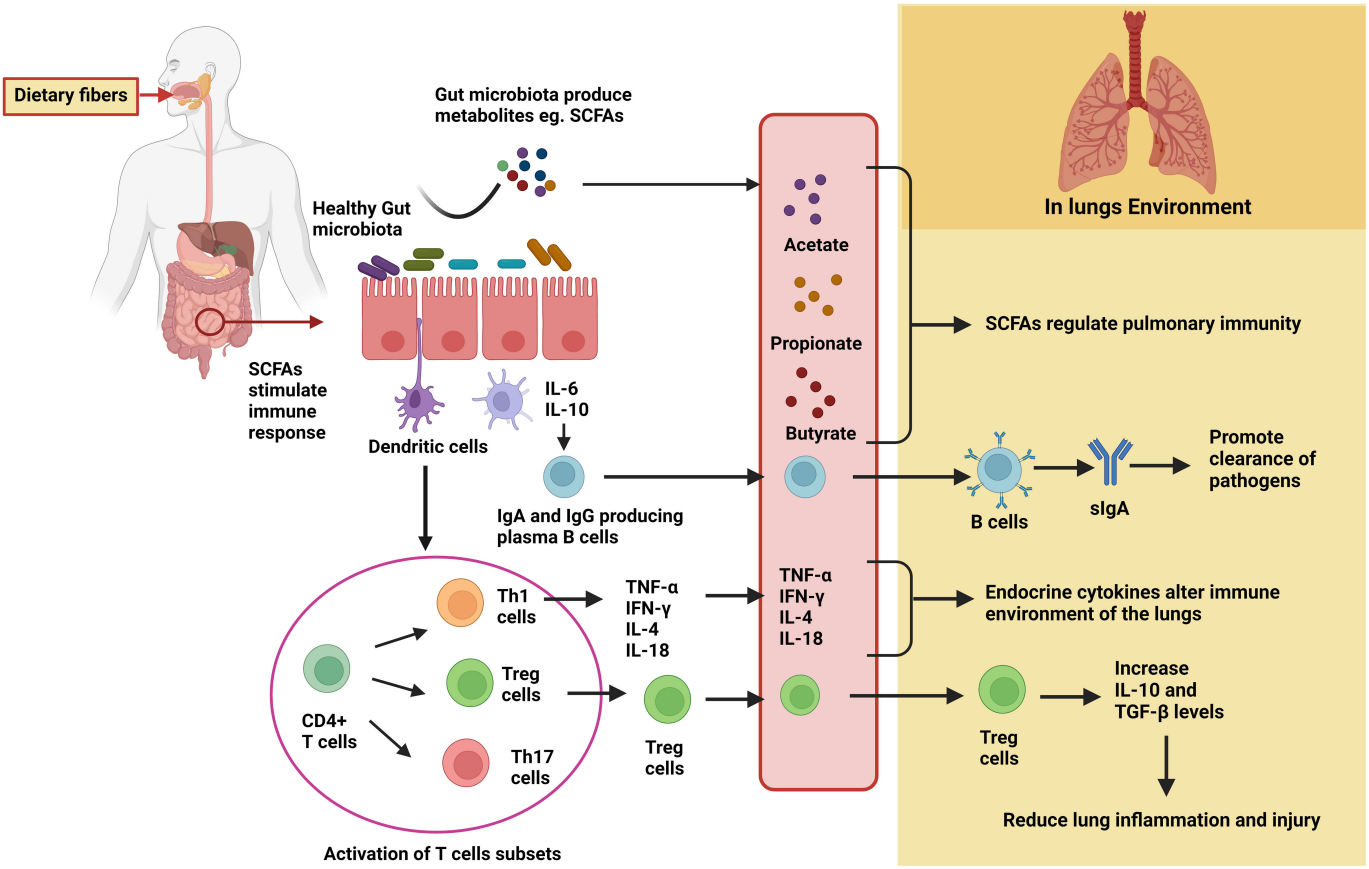
Detailed mechanisms of gut microbiota in modulating lung immunity through the gut–lung axis. Fermentation of dietary fibers by the gut microbiota results in the production of metabolites, particularly SCFAs. SCFAs either directly migrate to lung tissues through the circulation and regulate pulmonary immunity or promote differentiation and activation of immune cells to produce cytokines and IgA. In the lungs, IgA promotes clearance of pathogens, Treg cells reduce lung inflammation and injury, and certain cytokines (such as TNF-α, IL-4, etc.) alter the immune environment.

So far, the underlying mechanisms of SCFAs include two main signaling pathways. Most notably, the direct immune action of SCFAs is through the involvement of G protein-coupled receptors (GPCRs) (48). Among GPCRs, primarily GPR109A (NIACR1), GPR41 (free fatty acid receptor 2, FFAR3), and GPR43 (FFAR2) are differentially expressed by different cell types and tissues (48, 49). Following ligand binding, the GPCRs are coupled to distinct downstream effector molecules, i.e., G_i/o_ or Gq, thereby resulting in additional complexity to SCFA induced GPCR signal transduction, leading to different outcomes in cellular functions and responses in different cell types (50). Through this G protein receptor-based signal transduction, inflammatory responses are activated. The activation involves several protein kinases, such as phosphoinositide 3 (PI3K)-kinases, mitogen-activated protein (MAP) kinases, and mammalian target of rapamycin (mTOR) (51). Also, SCFAs modulate immune responses by downregulating the gene expression of histone deacetylase (HDAC) in different tissues (52). The suppressive effect of SCFAs is mediated by either diffusion or carrier/transporter proteins (H+-coupled carrier SLC16A1 proteins, Na+-coupled monocarboxylate transporter SLC5A8) (53). GPR43 is also involved in the suppression of colonic inflammation through the β-arrestin2 upregulation that impedes the activity of NF-κB. The opposing roles of GPR43 were discerned in the study where mice deprived of GPR43 activity were found to be susceptible to inflammation in the colon (53). Taken together, SCFAs perform pleiotropic functions in different cells and tissues. The SCFA ligands GPR109A and GPR43 and signaling proteins activate LRR, NACHT, and PYD domain-containing protein 3(NLRP3) inflammasome activation, which supports cellular repair mechanisms. Lack of NLRP3 inflammasome activation in mice leads to a higher susceptibility to developing colon cancers. Notably, dietary fiber-mediated colonic inflammation involves activation of NLRP3 accompanied by GPR43 signaling and K+ efflux (54). Another study demonstrated that chronic colitis induced mice lacking GPR43 were more resistant to developing colonic inflammation than the WT group (55). Considering the reasons for these conflicting reports, it can be deduced that the presence of diverse gut microbiota may be one of them. The two signaling proteins, GPR41 and GPR43, play a role in the clearance and/or colonization of some gut microbes, if not all, which in turn either suppress or promote inflammation in the colon (56). Also, another mechanistic approach of SCFAs in regulating inflammation is mediated by activation of colonic forkhead box P3 (Foxp3)^+^ Treg cell differentiation involving GPR43 signaling proteins (53). SCFAs modulate immune responses by upregulating the gene expression of histone H3 acetylation at the Foxp3 locus, bringing permissive chromatin structure that leads to greater gene accessibility for transcription. The recent findings stated the role in promoting gut homeostasis by upregulating fatty acid β-oxidation (FAO) pathway in colonic epithelial cells (53). There are several *in vitro* studies which have shown that the peroxisome proliferator-activated receptor gamma (PPARγ), which is a nuclear transcription factor, is induced by butyrate. The luminal anaerobiosis supports the viability of a butyrate-producing microbes, particularly belonging to the phyla *Bacteroidetes* and *Firmicutes*. Concomitantly, gut lumen hypoxia impedes the growth of dysbiotic microbes, largely facultative anaerobic bacteria belonging to the phylum Proteobacteria (54). Given their impact on the intestinal tract, SCFAs can also promote an accumulation of thymic peripheral Treg cells, which is associated with allergic airway diseases, particularly by acting as HDAC inhibitors. During respiratory infection caused by influenza, SCFAs serve as a substrate for FAO to increase cellular metabolism and function of CD8^+^ T cells. There is a vast literature available voicing and assuring the role of diet, microbiota, SCFAs, and immune response on the gut–lung axis, which are illustrated in **Figure 4**.

## Role of the gut microbes and metabolites on lung homeostasis

There are several studies available that confirm the role of gut microbiota in regulating immune responses at distal mucosal sites, particularly in the lungs (57). Both the gut microbes and their metabolites stimulate the mucosal immune responses at distal sites. Based on the functional properties and anatomy, the mucosal immune system acts both as an inductive site as well as an effector. The migration of cells from mucosal inductive site to effector site through the lymphatic system determine the mucosal immune responses in distant organs such as the gastro-intestinal tract, lung, etc. (57). The mucosal inductive sites form mucosa-associated lymphoid tissue (MALT), which is covered by microfold (M) cells that take up antigens present in the lumen of the intestinal mucosa and transfer them to dendritic cells (DCs), which in turn present antigens and initiate the mucosal T and B-cell responses. MALT is comprised of both nasopharyngeal associated lymphoid tissues (NALT) and gut-associated lymphoid tissues (GALT) (57). The GALT work as inductive sites and are comprised of organized lymphoid tissues (Peyer patches and mesenteric lymph nodes). Upon antigen exposure (like gut pathogens), GALT-DCs initiate differentiation of IgA-secreting plasma B cells (58). These differentiated IgA-producing plasma cells move out into the bloodstream and reach the gastrointestinal effector site (lamina propria) where they act against pathogenic microbes. Additionally, the gastrointestinal lamina propria region also contains plasma and memory CD4+ T helper cells, which promote the differentiation of IgA-producing B cells. The presence of gut microbiota is critical for controlling the induction and functioning of mucosal immunoglobulin A, as evidenced by studies where immunity and IgA production increase after colonization by gut commensals (58). As both induced T and B cells in Peyer’s patches move into the bloodstream and move to gastrointestinal and extra-intestinal sites like bronchial epithelium, it indicates the passing of “immunological information” between different organs. Also, bloodstream and/or lymph serve as connecting bridge between the gut (where primary sensitization occurs) and the affected site on the lung (58).

Soon after leaving the circulation, immune cells reside in mucosal effector regions in the body, such as the lung. In the lungs, they interact with high endothelial venules (HEVs). Interaction between adhesion molecules, VCAM-1 and α4β1 integrins as well as L-selectin and PNAd are required for the immune cell homing in lungs (59). The T effector memory (TEM) cells, which are formed in response to gut inflammation, express L-selectin/CD62L, which binds with PNAd ligands on the endothelial surface of the lungs as soon as they enter the systemic circulation (59). This demonstrates the migration of gut lymphocytes (IgA plasmablasts and TEMs) to lymphoid tissues of the lung. In line with the above-mentioned facts, it became apparent that the lung and gut together contribute to the mucosal immune system, and an inflammatory response in one of these organs may be reflected in the other organ as well.

The cellular components of the immune system homing in the gastrointestinal lamina propria and mesenteric lymph nodes are capable of neutralizing most of the translocating microbes (60). Most of the live microbes/pathogens as well as the fragments of dead bacteria move from the mesenteric lymphatic system and enter systemic circulation (61) where they can reach lungs and modulate immune response. Microbial metabolites like SCFAs, directly enter systemic circulation and affect lung. Interestingly, high-fiber-fed mice exhibited higher circulating SCFA levels and were found to be protected against allergic inflammation in the lungs, while low-fiber-fed mice showed lower circulating SCFAs and increased susceptibility to allergic pulmonary disease (62). To elucidate, higher SCFA levels are accompanied by a surge in dendritic cell differentiation and proliferation. Subsequently, dendritic cells seed the lungs with high phagocytic capacity and a reduced tendency to promote Th2 cell effector function (62).

## Lung microbes influence gut health

Although the mentioned literature dealt with the impact of a complex gut community on pulmonary immune response, the other side still remains unexplored and is a potential area of research. However, till date, only limited studies have established the impact of lung pathogenic microbes on gut health. Few studies involved murine models of influenza, where acute H1N1 and H5N1 influenza A viruses (IAVs) were reported to develop gastrointestinal symptoms, mainly diarrhea and vomiting, owing to gut dysbiosis (63–65). This perturbation of the gut microbiota due to respiratory IAV infection also applies to humans where H7N9 subtype virus infection significantly reduces diversity and enhances the growth of microbes, especially *Enterococcus faecium* and *Escherichia coli* (66). Influenza-associated dysbiosis was functionally explored and fully characterized by Sencio and coworkers (2020), who reported the changes in gut (intestinal and cecal) microbiota and subsequent reduction in short-chain fatty acids (SCFAs) production upon sublethal infection with the H3N2 and H1N1 subtypes of influenza in a murine model. In mechanistic terms, diminished SCFA production (predominately acetate) was attributed in part to decreased food consumption, which is a characteristic feature of influenza alterations in the microbiota that compromise respiratory immune response against infection. Supplementation with acetate during influenza infection supported lung defenses against secondary pneumococcal infection in a free fatty acid receptor 2 (FFAR2)-dependent manner, thereby augmenting the lethal outcomes. Moreover, pharmacological activation of SCFA receptor FFAR2 aped the acetate effects and imparted protection against post-influenza pathogenic superinfections (67).

In the lungs, bacterial pneumonia caused by multidrug-resistant *Pseudomonas aeruginosa* or *Staphylococcus aureus* induces gastrointestinal enteropathy (68, 69). Moreover, bacterial pneumonia infection specifically by *P. aeruginosa* has been reported to curtail gastrointestinal epithelial cell proliferation by restricting the M-phase of the cell cycle (58, 70). In order to understand the “lung–gut” axis wherein chronic lung inflammation disturbs the gastrointestinal and blood microbiota composition, an important study was conducted on C57BL/6 mice (71). Acute lung injury induced by an intra-tracheal single dose of lipopolysaccharide showed higher cecum bacterial load and a reduction in bacterial diversity and load in bronchoalveolar lavage fluid (BAL). Notably, a study showed dysbiosis in the airway microbiota is accompanied by an increase in the microbial load in the gut, ultimately leading to the disruption in gut microbial diversity. This gut dysbiosis was attributed to the translocation of bacteria from the lungs into the intestine through the blood (71). Taken together, the lung–gut axis acts as a bilateral loop that is significantly activated by alterations in lung or gut immune responses (61).

## Microbial metabolites interfere with SARS-CoV-2 infection

As already mentioned, microbial metabolites, specifically SCFAs in the gut, travel through the circulation and reach the lungs in a bilateral manner, mediating gut–lung axis crosstalk. Most notably, SCFAs from gut microbiota influence the bone marrow hematopoietic precursors, thereby reducing pulmonary inflammatory response. In light of that, it is plausible to assume that SCFAs may influence the host immune responses to viral infection, such as SARS-CoV-2. To date, according to the World Health Organization (WHO), SARS-CoV-2 has already infected more than 3.08 million people worldwide, with 5,492,595 deaths (https://covid19.who.int/). Growing evidence suggests that the SARS-CoV-2 not only targets the pulmonary tract but also infects the intestinal tract as found in the stomach, esophagus, duodenum, and in fecal samples from COVID-19 patients. High loads of replicating viruses were detected in biopsies from the gastrointestinal epithelial layer of the COVID-19 infected subjects (72). Persistent in the gut, SARS-CoV-2 causes diarrhea-like symptoms, which correspond to reduced compositional diversity and richness of the gut microbiota, delayed immune response, and viral clearance (73). Basically, gut microbiota and their metabolites modulate the gene expression of type I interferon receptors in pulmonary epithelial cells that secrete cytokines IFN-α and IFN-β on virus exposure, thereby impeding the replication of virus (74). In addition, signals derived from the gut microbiota also activate specific CD4+ and CD8+ T lymphocytes expressing pro-IL-18, pro-IL-1β, and NLRP3. This inflammasome activation leads to differentiation and migration of dendritic cells from the lungs to the draining lymph nodes after viral exposure (75). In antibiotic-treated mice, expression of IFN-gRI, MHC-I, CD86, and CD40 molecules in peritoneal macrophages is reduced during early response to viral infection, stating that gut microbiota signals the innate immune response prior to viral replication in the host (76). In brief, the gut–lung axis may influence the SARS-CoV-2 replication *via* 1) microbiota signals the migration of immune cells between mucosal surfaces, the gut and the lungs (77); 2) cytokines and growth factors produced in the gut mucosa reach the systemic circulation and act on mucosal tissues of the lungs in response to commensal microbiota (78); 3) the microbial metabolites, particularly SCFAs, are absorbed in the gut mucosa and attach to immune cell receptors in the pulmonary tract, thereby augmenting anti-viral responses in the lung by regulating mucosal immunity. This effect is known as “metabolic reprogramming” (78).

Pascoal et al., (79) used human intestinal biopsies and intestinal epithelial cells to investigate the impact of SCFAs in the infection by SARS-CoV-2. They did not observe any change in the entry or replication of SARS-CoV-2 in intestinal cells. Microbial metabolites had no effect on intestinal permeability for SARS-CoV2 related antigens and presented only minor effects on the production of anti-viral and inflammatory mediators. In contrast, Baradaran Ghavami et al., (80) discussed SCFA enhances the cellular amount of ATP molecules, acetyl-CoA, and plasma B-cell differentiation derived lipid biogenesis to induce IgAs and Neutralizing Antibodies (NAb) secretion against SARS-CoV-2 infection and modulate immune reactions. Similarly, Piscotta et al., (81) discovered small molecules using a cell-based SARS-CoV-2 infection assay. They screened culture broth extracts from a collection of phylogenetically diverse human-associated bacteria for the production of small molecules with antiviral activity. They purified three bacterial metabolites capable of inhibiting SARS-CoV-2 infection. These natural antiviral compounds exhibit structural and biological similarities to synthetic drugs that have been clinically examined for use against COVID-19.

## Concluding remarks

So far, recent progress in host–microbe interaction studies has accentuated the role of microbial metabolites in maintaining tissue and immune homeostasis. Among these commensal-derived metabolites, SCFAs were found to be pivotal signaling molecules that play a significant role in inflammatory and protective immune responses within the gut and in the lungs. The systemic and local SCFA concentrations largely rely on fiber intake in our diets and on the gut commensals that possess the capacity to ferment these fibers. Thus, the fibers in diet are responsible for fostering the healthy gut microbiome by outnumbering the *Bacteroides* that are capable of enhanced SCFA fermentation. Detailed understanding of the protective role of SCFA in gastrointestinal and pulmonary inflammatory diseases has allowed treating patients by modulating their diet. The mechanistic approach of SCFAs in regulating inflammation is mediated by multiple pathways such as activation of colonic forkhead box P3 (Foxp3)^+^ Treg cell differentiation, inducing peroxisome proliferator-activated receptor gamma (PPARγ), upregulating fatty acid β-oxidation (FAO) pathway in colonic epithelial cells as well as promoting an accumulation of thymic peripheral Treg cells which is associated with allergic airway diseases, particularly by acting as HDAC inhibitors. However, further detailed studies are needed in the future to ascertain the role of SCFAs in other chronic inflammatory conditions such as autoimmunity and cancer. Taken together, the concept of the gut–lung axis has gained tremendous attention with the discovery of gut microbiota-derived SCFAs in priming the immune system against allergic and infectious responses in the airway. The gut–lung axis may also influence the SARS-CoV-2 replication, which is mediated by SCFAs that are absorbed in the gut mucosa and tend to attach to immune cell receptors in the pulmonary tract, thereby augmenting the anti-viral response in the lung by regulating mucosal immunity. This mechanistic explanation holds great potential for future therapies.

## Author contributions

SR designed and wrote the manuscript. SM has contributed in writing some sections, added some more information during revision and thoroughly reviewed the manuscript. SS contributed to bibliographic analysis and reviewed the manuscript. PT thoroughly reviewed the manuscript. All authors contributed to the article and approved the submitted version.

## Funding

SR was supported by SERB, India; CSIR-IITR, India provided partial financial support for the PT lab; DBT, India provided a Ramalingaswami Fellowship Research Grant; and SERB, India provided a Startup Research Grant (SRG/2020/001101).

## Conflict of interest

The authors declare that the research was conducted in the absence of any commercial or financial relationships that could be construed as a potential conflict of interest.

## Publisher’s note

All claims expressed in this article are solely those of the authors and do not necessarily represent those of their affiliated organizations, or those of the publisher, the editors and the reviewers. Any product that may be evaluated in this article, or claim that may be made by its manufacturer, is not guaranteed or endorsed by the publisher.
